# Pressure driven polymorphic transitions in nanocrystalline Lu_2_O_3_, Tm_2_O_3_ and Eu_2_O_3_

**DOI:** 10.1038/s41598-023-42181-3

**Published:** 2023-10-13

**Authors:** Neha Bura, Ankit Bhoriya, Deepa Yadav, Srihari Velaga, Bal Govind, Jasveer Singh, Himanshu Kumar Poswal, Nita Dilawar Sharma

**Affiliations:** 1 CSIR- National Physical Laboratory, Dr. K. S. Krishnan Marg, New Delhi, 110012 India; 2 Academy of Scientific and Innovative Research (AcSIR), Ghaziabad, 201002 India; 3 High Pressure and Synchrotron Radiation Physics Division, Physics Group, HBNI, Bhabha Atomic Research Centre, Mumbai, 400085 India

**Keywords:** Condensed-matter physics, Chemical physics, Materials chemistry

## Abstract

The crystallite size of the materials considerably influences the material properties, including their compressibility and resistance to external forces and the stability of the crystalline structure; a corresponding study for which, so far, has been limited for the important class of nanocrystalline Rare Earth Sesquioxides (REOs). In the present study, we report the crystallographic structural transitions in nanocrystalline Rare Earth Oxides (REOs) under the influence of pressure, investigated via high-energy X-Ray Diffraction (XRD) measurements. The study has been carried out on three of the REOs, namely Lutetium oxide (Lu_2_O_3_), Thulium oxide (Tm_2_O_3_) and Europium oxide (Eu_2_O_3_) up to the pressures of 33, 22 and 11 GPa, respectively. The diffraction data of Lu_2_O_3_ and Tm_2_O_3 _suggests the occurrence of irreversible structural transitions from cubic to monoclinic phase, while Eu_2_O_3 _showed a transition from the cubic to hexagonal phase. The transitions were found to be accompanied by a collapse in the volume and the resulting Pressure–Volume (P–V) graphs are fitted with the 3rd order Birch-Murnaghan (BM) equation of state (EOS) to estimate the bulk moduli and their pressure derivatives. Our study establishes a qualitative relationship between the crystallite size and various material properties such as the lattice parameters, transition pressure, bulk modulus etc., and strengthens the knowledge regarding the behaviour of this technologically important class of materials.

## Introduction

Rare earth sesquioxides (REOs) have been an interesting area of study in past years due to their versatile technological and scientific significance. They have various applications in every field of science and find applications as coating materials to stabilize high voltage lithium layered oxide cathodes^1^, as well as in enviornmental barrier coatings^2^. They are also used as a doping materials in metal oxides to increase their photoctatlytic activity^3^, blended with various polymers to increase their gamma shielding capacity^4^. These materials are of strategic importance and find applications in solid oxide fuel cells^5^, nuclear security applications^6^, neutron absorbers^7^, laser crystals^8,9^, nuclear waste host materials^10^, chemical machine polishing^11,12^, light emitting devices^13^, rare earth magnets, control rods for fast breeder reactors^14^, radiation sheilding^10,15,16^, and catalysis^17^ etc. REO nanoparticles are also used for biomedical and dental applications^18–20^. Due to these numerous applications in all fields of science, it becomes necessary to study the behavior of these materials under varying conditions. 

Three polymorphic forms viz. cubic, monoclinic and hexagonal are usually exhibited by REOs. They exhibit these structures depending on their cationic radii. As we move from La to Lu in the periodic table, the cationic radii decrease and their atomic masses increase. Heavier sesquioxides exhibit cubic structure (C-type) having a space group *Ia-3* (206) and Z = 16 while the lighter ones are found to be in a hexagonal phase (A-type) with space group *P-3m1* and Z = 1. The medium cations i.e., Sm to Gd can exist in the monoclinic phase (B-type) having a space group *C2/m* and Z = 6. Furthermore, it is known that under the application of external forces, the materials undergo phase transitions as they try to adjust their structure to minimize the lattice energy and achieve equilibrium. The structural transition from cubic to monoclinic to hexagonal is accompanied by volume collapse. The hexagonal phase is known to have the least volume among all three phases. However, the volume change in the case of the transition from the cubic to monoclinic phase has a high value, and contrary to this, the transition from the monoclinic to hexagonal phase is accompanied by small changes in the volume^21^.

The structural transition studies in the REOs are an interesting area of research and a number of researchers have been working on the same. Jiang et al. studied the high-pressure behavior of the Eu_2_O_3_ using Angle Dispersive X-Ray Diffraction (ADXRD) and observed a cubic to hexagonal transition with a volume collapse of 9.6 %^22^. Yu et al. reported a phase transition from cubic to hexagonal in nano-Eu_2_O_3_ which starts at 9.3 GPa^23^. Our group also reported a phase transition from cubic to monoclinic in Eu_2_O_3_ having some content of the Eu_1-x_O^24^. Recently, we have also reported a cubic to hexagonal transition in pure Eu_2_O_3_ which initiated at 4.79 GPa and completed at 15 GPa^25^. The pure Eu_2_O_3_ shows a direct transition from the cubic to the hexagonal phase. Moreover, Lu_2_O_3_ and Tm_2_O_3_ have been reported to show transition from cubic to monoclinic phase. Sahu et al. reported a transition from the cubic to monoclinic phase in Tm_2_O_3_ at 7 GPa using the XRD studies^26^. A phase transition from cubic to monoclinic phase at 12 GPa has been reported by Irshad et al. using Raman as well as XRD measurements in Tm_2_O_3_^27^. A cubic to monoclinic phase transition in Lu_2_O_3_, which started at a pressure of 12.7 GPa and completed at 18.2 GPa has been reported by Jiang et al.^28^. Lin et al. observed that a cubic to monoclinic transition started at a pressure of 17 GPa in Lu_2_O_3_^29^. Our group has also studied the pressure behavior of Lu_2_O_3_ using Raman spectroscopy and found that the cubic phase is stable up to the pressure of 15.6 GPa^30^. However, there are discrepancies in the value of the transition pressures reported so far.

Further, nanocrystalline materials, owing to their nanometric size, have fascinating properties different than the bulk materials due to the large surface area and large surface to volume ratio. These aspects of the nanomaterials also affect the crystallographic transition pressures of the materials. In general, the transition pressure of the REO nanomaterials is found to be higher than their bulk counterpart^23,31^. Such a study has also been reported for CeO_2_^32^. From the mechanical point of view, this could be attributed to the Hall–Petch effect due to which, the grain boundaries in the material affect its yield strength and with the decreasing grain size the yield and flow stress required for continuous plastic deformations increase. However, targeted investigations of pressure driven transitions in nanocrystalline REOs still have much scope.

In this study, we present the pressure-driven structural changes investigated via variation in the diffraction peaks for three of the nanocrystalline REOs namely Eu_2_O_3_, Tm_2_O_3_ and Lu_2_O_3_ using the high-pressure XRD (HPXRD) study up to the pressures of 11 GPa, 22 GPa, and 33 GPa respectively. We discuss the influence of the crystallite size on various material properties such as bulk modulus, phase transitions pressure, etc., and attempt to establish a qualitative relation between them, which has been missing so far. Along with this, we also report the dependency of the lattice parameters on pressure.

## Experimental procedure

The REOs of purity 99.99% were used as the starting materials. The powder sample of the Lu_2_O_3_ was procured from Johnson Matthey, UK and powder samples of Tm_2_O_3_ and Eu_2_O_3_ were procured from Alfa Aesar respectively.

The HPXRD measurements were carried out on beamline 11 of Indus 2 synchrotron radiation facility at RRCAT, Indore, India. To carry out the measurements, Lu_2_O_3_ and Tm_2_O_3_ were loaded in Fe-gaskets with a hole size of about 80 µm along with a few ruby chips which were used as pressure calibrators. However, for Eu_2_O_3_, the Fe-gasket was used with the gold as a pressure calibrator. In all the HPXRD studies, a mixture of methanol and ethanol in a ratio of 4:1 was used as the pressure-transmitting medium. The hydrostatic limit for this pressure transmitting medium is 10.5 GPa^33^. The wavelength of the incident radiation was tuned at 0.696112 Å for both Lu_2_O_3_ and Tm_2_O_3_, and tuned at 0.634857 Å for the Eu_2_O_3_. The distance and orientation of the detector were calibrated using CeO_2_ powder as well as LaB_6_.

### Characterization at ambient

All the samples, in their ambient phase, were found to exhibit the cubic crystalline phase with space group *Ia-3(206)*. The various characterization studies including XRD, Raman spectroscopy, SEM and EDX measurements, carried out on these samples have been reported elsewhere in references^25,30,34^. The obtained diffraction patterns of the sesquioxides were compared with the JCPDS data cards, #430121 for Lu_2_O_3_, # 431034 for Tm_2_O_3_ and #340392 for Eu_2_O_3_. The crystallite size of all three materials, estimated using Williamson-Hall plot, was found to be in the range of 30–66 nm which confirmed the nanocrystalline nature of all the studied materials as mentioned in Table 1. The phase purity of the samples was also further confirmed by Rietveld analysis of the ambient XRD spectra of the REOs obtained using the synchrotron radiation source as shown in Fig. S1. The various lattice parameters and ambient unit cell volumes obtained from the refinement are also shown in Table 1.

**Table 1:** Lattice parameters and unit cell volume of the REOs.

Material	Lattice parameters (Å)			Unit cell volume (Å^3^)	Volume/Z (Å^3^)	Crystallite Size^25,34^
	a	b	c			
Eu_2_O_3_	10.8540 (2)			1278.71 (4)	79.92	66 nm
Tm_2_O_3_	10.4723 (3)			1148.48 (5)	71.78	35 nm
Lu_2_O_3_	10.3755 (1)			1116.92 (3)	69.81	30 nm

### Pressure-dependent XRD measurements and data analysis

#### Lutetium oxide (Lu_2_O_3_)

**Figure 1:**
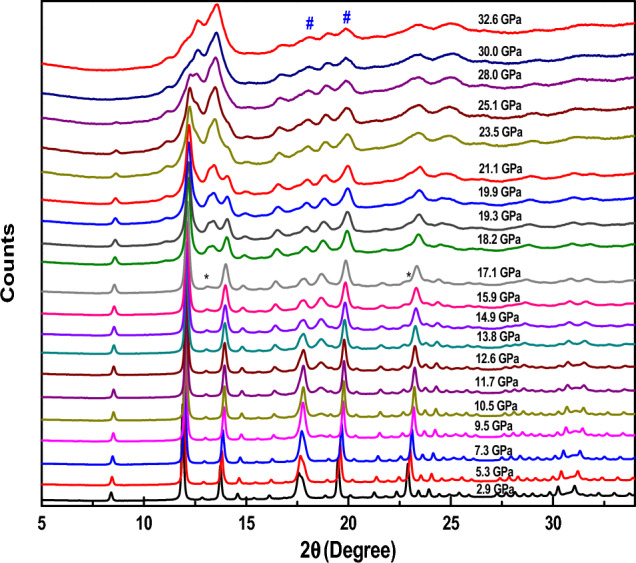
XRD patterns of Lu_2_O_3_ with increasing pressures. Each diffraction data is marked with the corresponding pressure and the peaks corresponding to the gasket material have been marked with #.

For Lu_2_O_3_, the pressure-dependent measurement was carried out up to a pressure of 33 GPa. Figure 1 shows the pressure-dependent XRD results. The XRD pattern at any given pressure contains the peak of the sample in question, as well as the peaks corresponding to the gasket material (Fe). As we increase the pressure, the peaks of the cubic phase start shifting towards the higher values of 2θ while simultaneously, peaks also start to broaden. Up to a pressure of 17.1 GPa, peaks only shift towards the higher 2θ side. However, at around 17.1 GPa new peaks started to develop at about 2θ = 13.45 º, which is marked with * in Fig. 1. In addition, a weak shoulder to the cubic phase peak at 2θ = 22.96 º also begins to develop. With further increasing pressure, the intensity of these peaks increases significantly at the expense of the intensity of the cubic phase peaks.

At around 30 GPa, the peaks corresponding to the cubic phase disappear entirely. The newly developed peaks agree very well with the monoclinic phase of the material which is confirmed by Rietveld refinement of the high-pressure data. Hence, we can say that a pressure-driven structural transition from the cubic to the monoclinic phase has been observed. This phase transition starts at 17.1 GPa of pressure and completes at 30 GPa. Beyond 30 GPa, the monoclinic phase of the material is found to be stable till the highest studied pressure i.e., 32.6 GPa.

#### Thulium oxide (Tm_2_O_3_)

**Figure 2:**
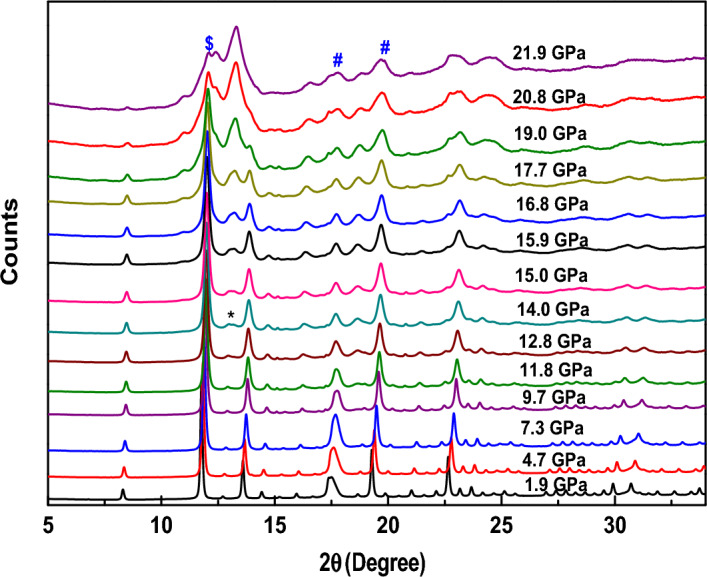
Evolution of the diffraction pattern of Tm_2_O_3_ with increasing applied pressure. Peaks of the gasket material, i.e., Fe are marked with #.

The pressure-driven changes can be seen in Tm_2_O_3_ up to the highest studied pressure of around 22 GPa in Fig. 2. Along with the peaks of the cubic phase of the sample, the peaks corresponding to the Fe have also been observed in the diffraction pattern. The cubic phase of the material is found to be stable till the pressure of 12.8 GPa. At a pressure of 14.0 GPa, new peaks started appearing in the diffraction pattern at 2θ values of 13.04° and 22.57°. Similar to the observation in Lu_2_O_3_, with increasing pressures, the intensity of these peaks is found to be increasing while the intensity of the cubic phase peaks decreases. These new peaks also agree very well with the peaks of the monoclinic phase as concluded by Rietveld refinement of the data. However, till the highest studied pressure the transition was not completed since the peak corresponding to the Miller indices (222) belonging to the cubic phase, can still be observed in the diffraction pattern marked by $ in Fig. 2.

#### Europium oxide (Eu_2_O_3_)

**Figure 3:**
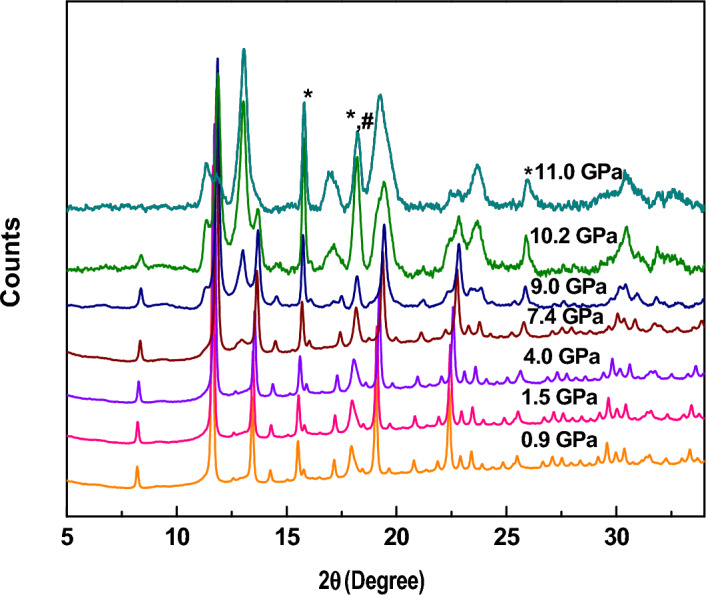
Pressure-driven changes in the diffraction pattern of Eu_2_O_3_. The peaks corresponding to Au (Pressure calibrator) and Fe (gasket material) have been marked with * and # respectively.

The structural changes in Eu_2_O_3_ with increasing applied pressure are shown in Fig. 3. With increasing pressure, the peaks were again observed to be shifted towards the higher values of 2θ, and simultaneously, the broadening of the peaks has been observed. Till the pressure of 4.0 GPa, we have only observed the broadening and shifting of the peaks. Nevertheless, at a pressure of 7.4 GPa, a new peak has been observed at 2θ = 12.7°, which is found to belong to the hexagonal phase of the sample, as confirmed by Rietveld refinement. With further increasing pressure, the intensity of this peak increases along with the emergence of additional new peaks. On the other hand, peaks corresponding to the cubic phase of the sample are losing their intensity, confirming the formation of a new phase at the cost of the current phase. However, till the highest studied pressure, the peaks corresponding to the cubic as well as hexagonal phases have been observed. Hence it is clear that up to a pressure of 11 GPa, the transition has not been completed.

### Data analysis

**Figure 4:**
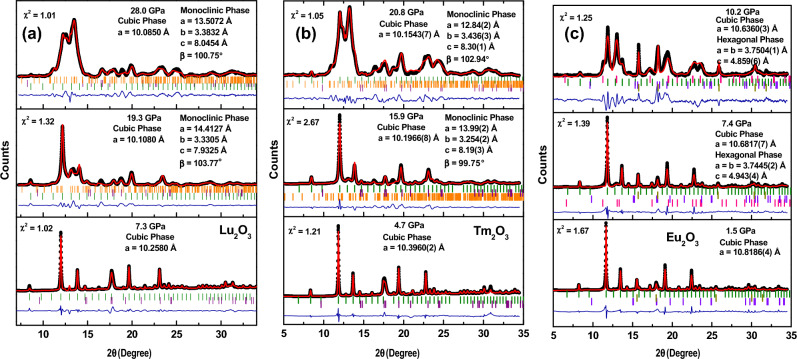
Rietveld refinement of (**a**) Lu_2_O_3_ and (**b**) Tm_2_O_3_ at a few applied pressures. The green and orange lines represent the Bragg’s positions corresponding to cubic and monoclinic phases respectively. (**c**) Rietveld refined diffraction data for Eu_2_O_3_ at various applied pressures. Green and pink ticks represent the Bragg’s positions corresponding to cubic and hexagonal phases of the Eu_2_O_3_ respectively. The dark yellow line represents the Bragg’s position of the gold and violet line represents the Bragg’s position of the Fe.

As mentioned, the Rietveld refinements at all the pressure points have been carried out for all the three samples using the CIF files available at Crystallographic Open Database (COD). For the refinement of the data, pseudo-Voigt peak shape has been used in the FullProf software. Rietveld refinement for all of three oxides at three selected pressure points is shown in Fig. 4. The values of the chi-square have been mentioned in the graphs which decides the goodness of the refinement. The values of the lattice parameters and volumes have been estimated using the refinement data.

**Figure 5:**
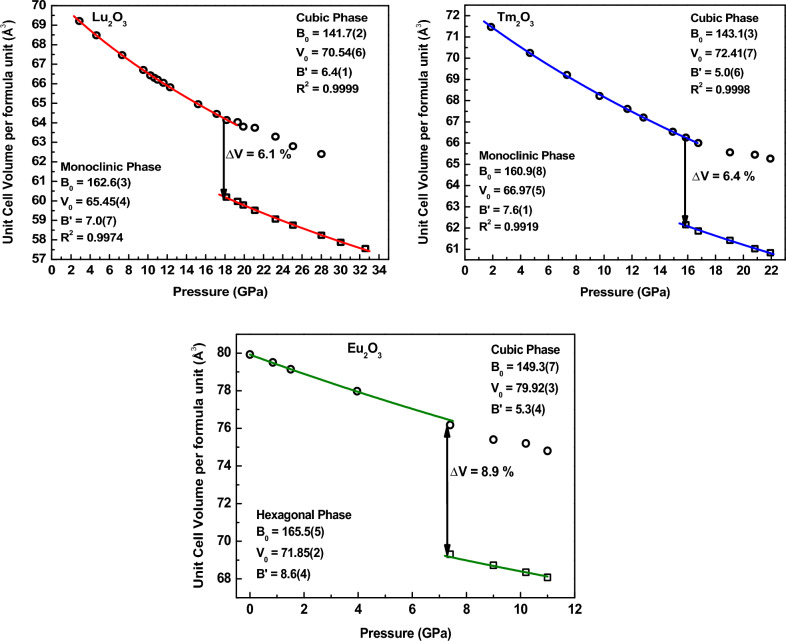
P-V plots for Lu_2_O_3,_ Tm_2_O_3_ and Eu_2_O_3_. The symbols represent the experimental data and solid line represents the fit using the BM EOS. Error in the volume is smaller than the symbol size.

The applied pressure affects the intermolecular bonding in the materials and this compression results in the decrease in the volume. As expected, we have also observed this decrease in the volume for all the samples with applied pressure as shown in Fig. 5. The squares and circles show the experimentally obtained pressure-volume data. All the three samples show significant reduction in volume near the transition pressures.

This pressure volume data was fitted using the BM EOS. However, prior to EOS fitting, the data was deduced from the F-f plot (Fig. S2). F represents the normalized stress and f is Eulerian strain and the estimations were carried out using the equations

1 $$F=\frac{P}{{3f\left(1+2f\right)}^{2.5}},$$

2 $$f=0.5\left[{\left(\frac{Vo}{V}\right)}^{2/3}-1\right].$$

The F-f plot comes out to be a straight line (Fig. S2), and hence 3rd order BM EOS has been used for the fitting of the obtained P-V data, which is given by

3 $$P\left(V\right)=\frac{3{B}_{0}}{2}\left[{\left(\frac{{V}_{0}}{V}\right)}^{7/3}-{\left(\frac{{V}_{0}}{V}\right)}^{5/3}\right]\left\{1+\frac{3}{4}\left({B}_{0}{\prime}-4\right)\left[{\left(\frac{{V}_{0}}{V}\right)}^{2/3}-1\right]\right\}.$$

In the above equation ${V}_{0}$ and V represent the unit cell volume of the material at ambient pressure and at pressure P respectively, ${B}_{0}$ is the bulk modulus of the elasticity and ${B}_{0}{\prime}$ is its pressure derivative.

The solid lines in Fig. 5 show the fit obtained for the experimental variation of the volume with pressure. In case of Lu_2_O_3_ and Tm_2_O_3_, the volume data is shown for the ambient cubic and the high-pressure monoclinic phase, while in case of Eu_2_O_3_ volume data is shown for the ambient cubic and the high-pressure hexagonal phase. From fitted data obtained using Eq. (3), the values of the bulk modulus and its pressure derivatives are estimated, which are given in Table 2 and mentioned in the graphs.

**Table 2:** Values of various parameters obtained from the fitting of the P-V graphs.

Material	Phase		${B}_{0}$ (GPa)	${B}_{0}{\prime}$	V_0_/Z (Å^3^)	Crystallite size
Lu_2_O_3_	Cubic	This work	141.7 (2)	6.4 (1)	70.54 (6)	30 nm
		Jiang et al.^28^	214 (6)	9 (1)	70.06	Bulk
		Lin et al.^29^	113.5 (7)	1.7 (3)	–	Pulverized crystals
	Monoclinic	This work	162.6(3)	7.0 (7)	65.45 (4)	30 nm
		Jiang et al.^29^	218 (13)	2.3 (3)	65.2 (2)	Bulk
Tm_2_O_3_	Cubic	This work	143.1 (3)	5.0 (6)	72.41 (7)	35 nm
		Irshad et al.^27^	149 (2)	4.8 (5)	72.10	112 nm
		Sahu et al.^26^	154.5	4 (fixed)	–	–
	Monoclinic	This work	160.9 (8)	7.6 (1)	66.97 (5)	35 nm
		Irshad et al.^27^	169 (2)	4 (fixed)	66.52	112 nm
Eu_2_O_3_	Cubic	This work	149.3 (7)	5.3(4)	79.92 (3)	66 nm
		Jiang et al.^22^	145 (2)	4 (fixed)	80.30 (3)	Bulk
		Yu et al.^23^	178(5)	4(fixed)	–	18 nm
	Hexagonal	This work	165.5 (5)	8.6 (4)	71.85 (2)	66 nm
		Irshad et al.^35^	165 (6)	4 (fixed)	71.35	–
		Jiang et al.^22^	151 (6)	4 (fixed)	74.4 (3)	Bulk
		Yu et al.^23^	229 (2)	4 (fixed)	–	18 nm

The table also compares the presently obtained values and the reported literature values and show a good agreement with the work of Irshad et al.^27,35^ for Tm_2_O_3_ and Eu_2_O_3_.

## Discussion

The predominance of surface atoms at the nanoscale, with their varied contributions to the cohesive energy through their related surface energies, can completely alter a phase energy landscape with a variety of effects. Due to this energy contribution, the phase diagram of the nanomaterial can change in comparison to its bulk counterpart in terms of the change in the transition pressures, involves new crystallographic structures or have amorphous states. In the present study, we have also observed a shift in the transition pressures in case of Lu_2_O_3_ and Tm_2_O_3_ towards higher value of pressure as compared to the reported studies^27–29^. However, in the case of Eu_2_O_3_, the transition pressure of 7.4 GPa, observed presently, is lower than the transition pressure reported by Jiang et al.^22^. Eu_2_O_3_ showed a transition to hexagonal phase while the other two samples progressed to monoclinic phase at high pressures.

The normal trend of the structural phase transitions in the REOs under the application of pressure is cubic to monoclinic and monoclinic to hexagonal^36–39^. However, exceptions have been observed in a number of oxides which show a direct transition from cubic to hexagonal transition^40–46^. This can be due to the lower energy of the hexagonal phase rather than the monoclinic phase^47^. In the present study, we have observed the normal trend of transition in the case of Lu_2_O_3_ and Tm_2_O_3_, but contrary to these results, Eu_2_O_3_ shows a transition from cubic to hexagonal phase, which is due to lower energy of hexagonal phase.

### Effect of crystallite size on transition pressure

In Lu_2_O_3_, the structural transition is initiated around the 17.1 GPa of pressure and completed at 30.0 GPa with the disappearance of all the peaks corresponding to the cubic phase. These results are found to be consistent with the transition pressure of 17 GPa, reported by Lin et al. in the crystals of Lu_2_O_3_^29^. The value of the transition pressure is also found to be consistent with the our previously reported high pressure Raman spectra of Lu_2_O_3_ in which the phase is found to be stable up to the pressure of 15 GPa^30^. However, the pressure at which the transition starts in bulk is 12.7 GPa as reported by Jiang et al., which is lower by a value of 5 GPa as compared to the nanocrystalline Lu_2_O_3_^28^.

In case of Tm_2_O_3_, also, the transition pressure is found to be higher than the theoretically predicted value of 8 GPa. The transition pressure also has a slightly higher value than the pressure reported by Irshad et al.^27^ for having a crystallite size of 112 nm estimated using the results from the refienement.

In Eu_2_O_3_, we have observed a new peak at around 7.4 GPa of pressure which is roughly in agreement with our previously reported Raman data in which the phase transition started around 4.79 GPa of pressure^25^. Although, we cannot exactly compare the starting pressure in the case of XRD data as pressure is increased manually in both cases, however, in both investigations, i.e., Raman and XRD studies, we have observed the transition from cubic to hexagonal phase. It may also be mentioned here that Raman investigations are more sensitive to structural changes and therefore, lattice disturbances prior to shifting to a new phase are more likely to be reflected earlier in the Raman measurements as compared to XRD measurements. Also, these results are in agreement with other researchers, in terms of the observation of the cubic to hexagonal phase transition^22,23^. Yu et al.^23^ reported a phase transition in the nano Eu_2_O_3_ having crystallite size of 18 nm which is less than our crystallite size and hence higher transition pressure for Eu_2_O_3_. Further, this observation is also consistent with our observation in case of Lu_2_O_3_ and Tm_2_O_3_. It is important to mention here that all the presently obtained results of the HPXRD data agree more or less with our previously reported results of high-pressure Raman investigations reported elsewhere^25,30^. In all these studies the mixture of methanol:ethanol in the 4:1 is used as the pressure tranmitting medium which has a hydrostatic pressure limit of 10.5 GPa^33^. As reported, non-hydrostatic pressure may cause deviatoric stresses and lead to significant broadening above the hydrostatic limit^48^. However, in the present exercise, we did not observe such an increase in the line-widths and therefore, deviatoric stresses, if present, apparently do not play a major role in determining the phase transition. This fact can also be supported by Eu_2_O_3_ data where the phase transition initiated below the hydrostatic limit.

In present case, the higher value of the transition pressure can be attributed to the smaller crystallite size of 30 nm and 35 nm in Lu and Tm oxides. Several researchers have reported similar behaviour for a number of other REOs^29,49^. Also, our group has earlier carried out studies on a number of other REOs and observed the higher stability of nanocrystalline materials^43–45,50–52^. It is therefore implied that the crystallite size plays a role in the transition paths and well as high pressure phases assumed by the nano-REOs. The higher stability of the Lu and Tm oxide nanomaterials can be attributed to the fact that polymorphic transition is part of solid–solid phase transformation where the nucleation usually occurs at the defect site in the lattice. However, in case of the nanocrystalline materials the defect density is limited due to which we need to overpressure the system to induce a phase transition. This over pressuring of the system explains the higher transition pressure in case of the smaller nanocrystalline materials. These observations are also consistent with the lower transition pressure observed for Eu_2_O_3_ with a larger crystallite size of 66 nm.

It is also worth mentioning that, as the number of f-electrons increase, the ionic radii of the lanthanides decreases which is also known as the lanthanide contraction. And due to this contraction, the stability of structure increases and as a result higher pressure is needed to change the structure. In our case, Eu has the highest ionic radius followed by Tm and then Lu, which suggests a lower transition pressure for Eu and highest for Lu. This agrees very well with our experimental results. This also agrees with our previously reported results for other REOs wherein the transition pressure was found to increase with decrease in the ionic radii^53^.

Hence, from the above discussion, the transition trend in the REOs remains the same; however, if we compare the onset pressure and pressure at which transition is completed, the values for nanocrystalline materials are higher than their bulk counterparts. Therefore, it may be surmised that transition pressure has an inverse relationship with the crystallite size. As mentioned, in all the presently investigated nanocrystalline materials, we have observed a change in the transition pressure as compared to their reported bulk counterparts and observed that smaller nanocrystalline materials show higher stability as compared to bulk materials^23,25–28,44,46,51,54–57^ and hence higher transition pressure.

**Figure 6:**
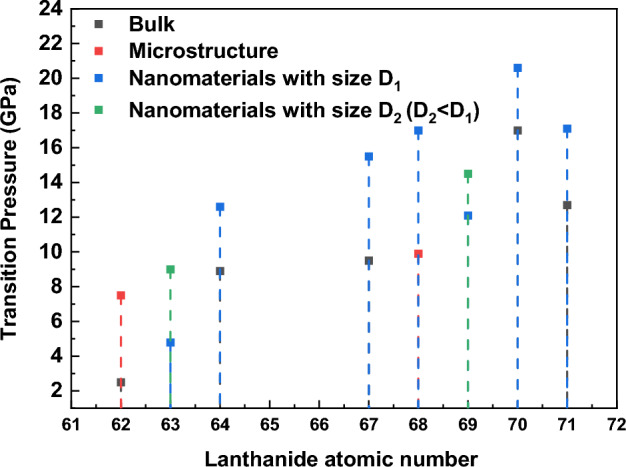
Transition pressure dependence on the crystallite size for various rare earth oxides^23,25–28,45,47,51,54–57^.

A comparison of the reported data is shown graphically in Fig. 6, along with the data of various other oxides. It is evident from the figure that bulk materials have the lowest transition pressure in comparison to their microstructured and nanocrystalline materials. Also, it is important to mention that nanomaterials having lower crystallite size show higher transition pressure.

### Effect of nanocrystalline nature of materials on bulk modulus

However, the results of the bulk modulus for the nanocrystalline materials are not as straightforward. It is found that the value of the bulk modulus for nanocrystalline materials is found to be lower than their bulk counterpart for some materials^40,49^, and contrary to this, it is found to be higher for some^23,56,57^. Some other oxides such as *In*_*2*_*O*_*3*_^58^, *Al*_*2*_*O*_*3*_^59^*, TiO*_*2*_^60^*, SnO*_*2*_^61^ also show a decrease in the bulk modulus with the decrease in the crystallite size.

As mentioned, the competition between the larger surface area and limited density of storage defects can play an important role in deciding compressibility of the materials. Due to the limited density of this defect storage, most of the nanoparticles (size less than the critical size (10–30 nm)) are defect free and hence show greater stability with external stimuli and perturbations and demonstrate higher compressibility which in turn leads to the lower value of the bulk modulus^49^. As discussed earlier, the higher value of transition pressure can be attributed to the fact that the phase transitions usually start from the defective region.

On the contrary, Yu et al. reported a higher value of the bulk modulus for the nanocrystalline Eu_2_O_3_ having crystallite size of 18 nm calculated using the Debye Scherrer equation in comparison to the bulk Eu_2_O_3_^23^.

Jiang et al. found the value of bulk modulus of Sm_2_O_3_ to be 149 GPa, when B_0_’ is fixed at 4^62^. However, for the submicron Sm_2_O_3_ procured from Acros, Guo et al. reported lesser value of the bulk modulus i.e., 142 GPa^40^. Yan et al. calculated the bulk modulus for the nano crystalline Ho_2_O_3_ having crystallite size of 14 nm estimated using Debye-Scherrer equation, which was 10 % lower than their bulk counterpart^49^. It is worth mentioning here that the higher value of compressibility of nano crystals is due to the surface effects. As the size increases, the contribution of these surface effects decreases, leading to lower compressibility and hence higher bulk modulus. In case of micron size structure e.g., for crystallite size in micrometre for Er_2_O_3_, Guo et al. reported a value of 200 GPa and 8.4 for the bulk modulus and first derivative of the bulk modulus respectively^57^. On the other hand, for the nanocrystalline Er_2_O_3_ the value for the B_0_ and B’ is found to be 181 and 4.07 respectively by Ren et al. which is less than submicron Er_2_O_3_^56^.

Our results are consistent with these results as the value of bulk modulus for the nanomaterials is lower than those reported for the bulk materials for Lu_2_O_3_ and Tm_2_O_3_. However, in case of Lu_2_O_3_, Lin et al. have reported the values which are lower than our values which can be due to the fact that the pulverized crystal has been used for the present studies^29^. In case of Tm_2_O_3_, results by the Irshad et al. are only slightly higher than the reported values which is expected as the study was carried out on the material having a crystallite size of 112 nm^27^.

It has been reported that with an increase in the atomic number, the value of the bulk modulus increases for the bulk REOs^26^. However, our present results neither endorse nor refute this statement since the bulk modulus values for the starting cubic phases, although marginally higher for Eu_2_O_3_ with larger crystallite size, were found comparable when considering the errors mentioned in the parenthesis. In addition, the values may not be directly compared considering the difference in the nano-size of the samples under investigation.

## Conclusions

The present investigations show that smaller nanocrystalline REOs show higher stability as compared to their bulk counterparts as well as larger nano-crystallites. The nano-size profoundly affects the transition pressures as well as the bulk modulus. We observed structural phase transitions in nanocrystalline Lu_2_O_3_, Tm_2_O_3_ and Eu_2_O_3_ under the application of pressure. Transition from cubic to monoclinic phase has been observed for Lu_2_O_3_ and Tm_2_O_3_, whereas transition from cubic to hexagonal phase has been observed for Eu_2_O_3_ due to lower energy of hexagonal phase. The results obtained using high-energy synchrotron XRD studies are consistent with previously reported high-pressure Raman results. The bulk modulus for all the nanocrystalline REOs have been reported which have values lower than their bulk counterparts.

### Supplementary Information

Supplementary Figures.

## Data Availability

The datasets generated and/or analysed during the current study are available in the COD repository, https://www.crystallography.net/cod/result.php.
