# Supplementary material for: Pressure driven polymorphic transitions in nanocrystalline Lu2O3, Tm2O3 and Eu2O3
Source: Sci Rep. 2023 Oct 13;13:17365. doi: 10.1038/s41598-023-42181-3 (PMC10575879; doi:10.1038/s41598-023-42181-3)
Supplement: Supplementary file 1 — Supplementary Figures. [file 41598_2023_42181_MOESM1_ESM.docx]

**Supplementary Information**

**Pressure driven polymorphic transitions in nanocrystalline Lu_2_O_3_, Tm_2_O_3_ and Eu_2_O_3_**

Neha Bura^1,2^, Ankit Bhoriya^1,2^, Deepa Yadav^1,2^, Velaga Srihari ^3^, Bal Govind^1,2^, Jasveer Singh^1^, Himanshu K Poswal^3^, Nita Dilawar Sharma^1,2*^

1. *CSIR- National Physical Laboratory, Dr. K. S. Krishnan Marg, New Delhi-110012, India.*
2. *Academy of Scientific and Innovative Research (AcSIR), Ghaziabad, 201002, India.*
3. *High Pressure & Synchrotron Radiation Physics Division, Physics Group, HBNI, Bhabha Atomic Research Centre, Mumbai 400085, India*

***[*ndilawar@nplindia.org*](mailto:ndilawar@nplindia.org)

**Figure S1: XRD pattern of the Eu_2_O_3_, Tm_2_O_3_ and Lu_2_O_3_ at ambient conditions obtained using the synchrotron radiation wavelength mentioned in the graph and corresponding Rietveld refinement data. In the Rietveld refinement graph the black circles, red line, pink tick marks, and the purple lines represent the experimental data, refined data, Bragg’s position and their difference respectively**

**Figure S2: F-f plot for PV data of Rare Earth Sesquioxide**
